# Prevention for those who have freedom of choice – or among the choice-disabled: confronting equity in the AIDS epidemic

**DOI:** 10.1186/1742-6405-3-23

**Published:** 2006-09-25

**Authors:** Neil Andersson

**Affiliations:** 1 Centro de Investigación de Enfermedades Tropicales, Universidad Autónoma de Guerrero, Apartado 2-25, Acapulco, Mexico

## Abstract

With the exception of post-exposure prophylaxis for reported rape, no preventive strategy addresses the choice disabled – those who might like to benefit from AIDS prevention but who are unable to do so because they do not have the power to make and to act on prevention decisions. In southern African countries, where one in every three has been forced to have sex by the age of 18 years, a very large proportion of the population is choice disabled. This group is at higher risk of HIV infection and unable to respond to AIDS prevention programmes; they represent a reservoir of infection. Reduction of sexual violence would probably decrease HIV transmission directly, but also indirectly as more people can respond to existing AIDS prevention programmes.

## Background

AIDS prevention in southern Africa serves those who can choose their HIV risks. Promoting abstinence [1], male or female condom use [2,3], microbicides [4] or reduced concurrency [5,6] all presume that beneficiaries will be choice-enabled. Male circumcision [7], quintessentially for choice-enabled males, does not address prevention for those who are coerced to have sex, female or male.

Victims of sexual abuse make up a big part of the southern Africa population. One in every ten – males and females – is sexually abused every year and one in every three has suffered sexual abuse by the age of 18 years [8]. With the exception of post-exposure prophylaxis for reported rape, no preventive strategy addresses these, the choice disabled, who might like to benefit from prevention but who are unable to do so because they do not have the power to make and to act on prevention decisions.

### Reservoir of infection

If the shortage of prevention approaches for the choice disabled is an equity oversight, it is a singularly dangerous one. The physical risk of HIV infection to victims is increased by lack of lubrication and trauma [9,10]. Champion reported an STI rate of 47% among sexual violence victims compared with 30% in the rest of the population from which they were drawn [11]. HIV prevalence rates are much higher among young women than men: 16% compared with 5% in one South African study [12]. In another, intimate partner violence and high levels of male control in a woman's current relationship were significantly associated with HIV infection [13]. In fact dozens of studies have found HIV risk factors associated with sexual coercion and that HIV-infected people experience more sexual coercion than those who are HIV-negative [14]. But these are nearly all cross sectional studies, making it impossible to conclude that sexual violence causes HIV infection.

Even so, however one looks at it, victims of sexual violence are a reservoir for infection that is not reached by existing prevention initiatives.

### Culture of sexual violence

The world view that goes with forced sex – inherently disdainful of others and their rights – contributes to the AIDS epidemic in other ways, like not disclosing one's HIV status to a sexual partner or refusing to negotiate condom use.

Our national survey of South African schools produced worrying findings about the culture associated with sexual violence. Children who suffered forced sex were very much more likely to believe they were HIV positive and less likely to be willing to go for testing. And children who had endured sexual abuse or who believed they were HIV positive were more likely to say they would spread HIV intentionally (20% among those who believed they were infected compared with 13% who did not believe so^8^).

Sexual abuse also affects the way survivors interpret education that attempts to reduce their risks [15].

### Downstream and side effects

AIDS prevention has downstream effects on HIV infection and negative secondary effects for the choice disabled. The only published RCT of male circumcision reported significantly more sexual contacts in the intervention group [7]. This could mean an increased risk of other STIs, including hepatitis. In a climate where millions of people are desperate for a solution to AIDS, protecting only choice enabled men gives out an unhelpful message.

Voluntary counselling and testing seems to produce irresponsible behaviour for some who test HIV-negative, despite protective effects behaviour change of those who test positive [16].

### Inefficient prevention investment

AIDS prevention limited to the choice enabled wastes investment. For example, the Gauteng provincial government in South Africa distributes around 100 million free condoms every year. For victims of sexual violence, however, condoms are not usually and option. The main impact of an apparently protective intervention, like male circumcision, will be for HIV-negative young men who are *not *victims of forced sex. If two in every ten are already HIV-positive and three in ten have been victims of sexual violence, this limits drastically the pool who can gain from male circumcision.

### Foundation for an epidemic

Forced sex is not the only factor in HIV infection but it is a factor we must deal with.

What would it take to prove that reducing sexual violence would reduce HIV infection – at least in a way that draws governments and donors to invest in this preventive strategy? It is impossible to monitor the sexual encounter where infection occurs. Cross sectional and even longitudinal studies cannot make the case. The only way to prove that reducing sexual violence reduces the risk of HIV infection is through randomised controlled trial where the intervention is to reduce sexual violence.

Even if reducing forced sex does not reduce HIV risks, the gain would still be considerable [17]. In the best of cases, we might reduce *both *forced sex and HIV risk.
